# Supplementary material for: HIVprotI: an integrated web based platform for prediction and design of HIV proteins inhibitors
Source: J Cheminform. 2018 Mar 9;10:12. doi: 10.1186/s13321-018-0266-y (PMC5845081; doi:10.1186/s13321-018-0266-y)
Supplement: Supplementary file 2 — Additional file 2. Source code of HIVProtI web server. [file 13321_2018_266_MOESM2_ESM.zip › HIVprotI_Source-code/hivprotI/weka-3-6-6/doc/weka/classifiers/rules/Rule.html]

Rule


---


|  |  |  |
| --- | --- | --- |
| **Overview** \| **Package** \| **Class** \| **Tree** \| **Deprecated** \| **Index** \| **Help** \| **Weka's home** | |  |
| **PREV CLASS**   **NEXT CLASS** | **FRAMES**    **NO FRAMES**     **All Classes** |  |
| SUMMARY: NESTED \| FIELD \| CONSTR \| METHOD | DETAIL: FIELD \| CONSTR \| METHOD |  |


---


## weka.classifiers.rules Class Rule

```
java.lang.Object
  weka.classifiers.rules.Rule
```

**All Implemented Interfaces:**: java.io.Serializable, Copyable, RevisionHandler, WeightedInstancesHandler

---

``` public abstract class Rule extends java.lang.Object implements WeightedInstancesHandler, Copyable, java.io.Serializable, RevisionHandler ```

Abstract class of generic rule

**Version:**
:   $Revision: 1.8 $

**Author:**
:   Xin Xu (xx5@cs.waikato.ac.nz)

**See Also:**: Serialized Form

---

**Constructor Summary**

`Rule()`


| **Method Summary** | |
| --- | --- |
| `java.lang.Object` | `copy()`             Get a shallow copy of this rule |
| `abstract  boolean` | `covers(Instance datum)`             Whether the instance covered by this rule |
| `abstract  double` | `getConsequent()`             Get the consequent of this rule, i.e. |
| `abstract  void` | `grow(Instances data)`             Build this rule |
| `abstract  boolean` | `hasAntds()`             Whether this rule has antecedents, i.e. |
| `abstract  double` | `size()`             The size of the rule. |

**Methods inherited from class java.lang.Object**

`equals, getClass, hashCode, notify, notifyAll, toString, wait, wait, wait`

**Methods inherited from interface weka.core.RevisionHandler**

`getRevision`

**Constructor Detail**

### Rule

```
public Rule()
```


**Method Detail**

### copy

```
public java.lang.Object copy()
```

:   Get a shallow copy of this rule

    :   **Specified by:**: `copy` in interface `Copyable`
    :   **Returns:**: the copy

---


### covers

```
public abstract boolean covers(Instance datum)
```

:   Whether the instance covered by this rule

    :   **Parameters:**: `datum` - the instance in question **Returns:**: the boolean value indicating whether the instance is covered by this rule

---


### grow

```
public abstract void grow(Instances data)
                   throws java.lang.Exception
```

:   Build this rule

    :   **Parameters:**: `data` - the data used to build the rule **Throws:**: `java.lang.Exception` - if rule cannot be built

---


### hasAntds

```
public abstract boolean hasAntds()
```

:   Whether this rule has antecedents, i.e. whether it is a default rule

    :   **Returns:**: the boolean value indicating whether the rule has antecedents

---


### getConsequent

```
public abstract double getConsequent()
```

:   Get the consequent of this rule, i.e. the predicted class

    :   **Returns:**: the consequent

---


### size

```
public abstract double size()
```

:   The size of the rule. Could be number of antecedents in the case
    of conjunctive rule

    :   **Returns:**: the size of the rule


---


|  |  |  |
| --- | --- | --- |
| **Overview** \| **Package** \| **Class** \| **Tree** \| **Deprecated** \| **Index** \| **Help** \| **Weka's home** | |  |
| **PREV CLASS**   **NEXT CLASS** | **FRAMES**    **NO FRAMES**     **All Classes** |  |
| SUMMARY: NESTED \| FIELD \| CONSTR \| METHOD | DETAIL: FIELD \| CONSTR \| METHOD |  |


---
